# Metal Oxide (Co_3_O_4_ and Mn_3_O_4_) Impregnation into S, N-doped Graphene for Oxygen Reduction Reaction (ORR)

**DOI:** 10.3390/ma13071562

**Published:** 2020-03-28

**Authors:** Penny Mathumba, Diana M. Fernandes, Renata Matos, Emmanuel I. Iwuoha, Cristina Freire

**Affiliations:** 1 Department of Chemistry, Faculty of Science, University of the Western Cape (UWC), Cape Town, Bellville 7535, South Africa; mathumbapz@gmail.com (P.M.); eiwuoha@uwc.ac.za (E.I.I.); 2 REQUIMTE/LAQV, Departamento de Química e Bioquímica, Faculdade de Ciências, Universidade do Porto, 4160-007 Porto, Portugal; renata.matos@fc.up.pt (R.M.); acfreire@fc.up.pt (C.F.)

**Keywords:** oxygen reduction, carbon material, metal oxides, heteroatom doping

## Abstract

To address aggravating environmental and energy problems, active, efficient, low-cost, and robust electrocatalysts (ECs) are actively pursued as substitutes for the current noble metal ECs. Therefore, in this study, we report the preparation of graphene flakes (GF) doped with S and N using 2-5-dimercapto-1,3,4-thiadiazole (S_3_N_2_) as precursor followed by the immobilization of cobalt spinel oxide (Co_3_O_4_) or manganese spinel oxide (Mn_3_O_4_) nanoparticles through a one-step co-precipitation procedure (Co/S_3_N_2_–GF and Mn/S_3_N_2_–GF). Characterization by different physicochemical techniques (Fourier Transform Infrared (FTIR), Raman spectroscopy, Transmission Electron Microscopy (TEM) and X-ray Diffraction (XRD)) of both composites shows the preservation of the metal oxide spinel structure and further confirms the successful preparation of the envisaged electrocatalysts. Co/S_3_N_2_–GF composite exhibits the best ORR performance with an onset potential of 0.91 V vs. RHE, a diffusion-limiting current density of −4.50 mA cm^−2^ and selectivity for the direct four-electron pathway, matching the results obtained for commercial Pt/C. Moreover, both Co/S_3_N_2_–GF and Mn/S_3_N_2_–GF showed excellent tolerance to methanol poisoning and good stability.

## 1. Introduction 

The continuous exhaustion of fossil fuels highly contributing to environmental problems and the increasing demand of energy has prompted the development of sustainable alternative energy sources and conversion devices like metal–air batteries and fuel cells. The major problem for the wide spread of these new technologies is related to the fact that the reaction occurring at the cathode, the oxygen reduction reaction (ORR), has sluggish kinetics and is the major rate-limiting factor restricting achieving the expected performance [1]. Ideally, this reaction should occur through a direct four-electron process, but for most of the electrocatalysts developed it occurs through a less efficient two-electron process with the production of an intermediate—hydrogen peroxide.

Currently, the most efficient electrocatalysts for ORR are noble metal platinum (Pt)-based materials with low overpotential, large current densities and selectivity toward the direct four-electron process [2,3]. However, these are extremely expensive due to limited platinum resources on earth, and they suffer deactivation due to methanol poisoning [3]. Consequently, the development and application of fuel cells and metal–air batteries have been limited. These drawbacks have stimulated the search for alternative, cost-effective and stable ORR electrocatalysts.

Among the most promising electrocatalysts to displace the expensive Pt/C are the earth-abundant first row transition metal nitrogen-doped carbons. These have gained particular attention since 1964, when Jasinski first observed ORR activity on a cobalt macrocyclic complex [4,5]. Cobalt and manganese oxides have emerged as a promising group of non-precious metal electrocatalysts due to their intrinsic electrocatalytic activity attributed to their characteristic mixed-valence states, high stability, low environmental impact, and exceptional 3d electronic configurations [6,7,8,9]. However, the limited number of active sites and the poor electrical conductivity have limited their wide application [10]. This drawback can be circumvented by designing hybrid materials of metal oxides supported onto electrically conductive materials. Carbon materials have been proven to modulate the electrical conductivity of cobalt, for example, by providing increased charge transport and exposing active sites due to the high surface area of carbon, thereby promoting the electrocatalytic activity of cobalt [11,12]. However, some studies have reported a limitation in electron transfer at the interface and restricted electrocatalytic tunability caused by the lack of bridged bonds between the carbon lattice and metal oxide, commanding the use of doped carbon to create effective bonds [13,14]. Graphene-based transition metal oxide (Fe, Co, Cu, Ni, and Mn) nanocomposites have already been a subject of study and have been proven to be a promising type of highly efficient and economic nanocatalyst for optimizing the ORR to solve the current energy crisis [11,15]. For example, iron oxides supported on N-doped carbon catalysts have been studied for a long time for the ORR due to the extremely low cost of Fe and their good performance in both acidic and alkaline electrolytes [16]. Similarly, different researchers reported the synthesis of cobalt oxides/N-graphene for ORR with very good electrocatalytic performances [17].

With this in mind, here we report the preparation of graphene flakes doped with nitrogen and sulfur (S_3_N_2_–GF) to serve as bridging atoms to facilitate electron transport. Subsequently, two composites based on the immobilization of Co_3_O_4_ and Mn_3_O_4_ nanoparticles supported on S_3_N_2_–GF were developed and efficiently employed for oxygen reduction. A scalable and easy procedure was used for the N- and S-doping process which constitutes an advantage. Additionally, the designed ECs exhibited enhanced ORR performances as a result of the covalent coupling effect between the S- and N-doped GF and the metal oxide nanoparticles.

## 2. Materials and Methods 

### 2.1. Materials and Instrumentation 

The materials and solvents used in the electrocatalyst preparation and in the electrocatalytical studies were used as received and are described in detail in the Supplementary Material (SM) file. All electrocatalysts were characterized prior to their application using different techniques (Raman, FT-IR, XRD, XPS and TEM) and the methods and equipment used are detailed in the SM file.

Electrocatalytic performance was evaluated using a PGSTAT 302N potentiostat (Metrohm Autolab B.V., Utrecht, The Netherlands) controlled by NOVA 2.1 and using a conventional 3-electrode system. For all details regarding electrodes, electrode conditioning and modification, see the SM file. 

### 2.2. Synthesis of S, N-graphene Flakes (S_3_N_2_–GF)

The preparation of dual-doped GF (nitrogen and sulfur) was achieved using a precursor containing both heteroatoms (2-5-dimercapto-1,3,4-thiadiazole, S_3_N_2_). Briefly, 400 mg of GF was mixed with 400 mg of S_3_N_2_ in a ball miller (Retsch MM200, Retsch GMBH, Haan, Germany) at 15 Hz for 5 hours. For a proper mixing of the two components, were chosen balls of zirconium oxide with a 2 mm diameter (≈100). Then, the mixture was subjected to calcination at 800 ºC for 1 hour under N_2_ flow. 

### 2.3. Synthesis of Co and Mn/S_3_N_2_–GF

For Co/S_3_N_2_–GF, the S, prior-prepared N-doped GF (235 mg) were dispersed in aqueous solution (50 mL) containing 4 mmol of CoCl_2_.6H_2_O. Then, the solution of 1-amino-2-propanol (MIPA) (3 mol dm^−3^) was added, at a rate of 50 mL h^−1^, until the pH = 10. This mixture was stirred at room temperature during 24 h and the slurry material was filtered, washed with water and ethanol and left to dry 12 h under vacuum (at 50 °C). Finally, the obtained material was calcined at 250 °C for 3 h in air. For the preparation of Mn/S_3_N_2_–GF, the procedure was very similar: 217 mg of S_3_N_2_–GF and 4.4 mmol MnCl_2_.4H_2_O were used, and calcination with air flux for 5 h at 300 °C.

## 3. Results and Discussion

### 3.1. Electrocatalysts Characterization

The successful doping of graphene flakes was confirmed by X-ray photoelectron spectroscopy (XPS) analysis and the high-resolution spectra are depicted in Figure S1. The C 1s high-resolution spectrum of S_3_N_2_–GF was fitted with six peaks at: 283.0 (C–S–C), 284.6 eV (sp^2^, C–C, C=C), 286.1 eV (C-O, C–N), 287.1 eV (C=O), 288.3 eV (O–C=O), and 290.7 eV (π-π* transition) [18,19]. The fitting was similar to that of pristine GF published previously with the exception of a new small peak corresponding to C–S–C (from doping with S_3_N_2_) and the contribution of C–N bonds to the peak at 286.1 eV [18]. The O 1s high-resolution spectrum of S_3_N_2_–GF is shown in Figure S1b and was fitted with three peaks: one at ≈ 531.3 eV corresponding to C=O, one at ≈ 532.8 eV attributed to C-O and another at ≈ 534.6 eV assigned to O-C=O [18,20]. The N 1s XPS spectrum of S_3_N_2_–GF (Figure S1c) was fitted with three main peaks at 398.4, 399.8 and 400.7 eV, attributed to pyridiniC–N, pyrroliC–N, and graphitic N, respectively. Those at 398.4 and 399.8 eV may be attributed to the π-conjugated system with a pair of p-electrons in graphene layers, while those at 400.7 eV demonstrate the replacement of N atoms in the carbon layers [21,22]. The S 2p XPS spectrum (Figure S1d) is slightly complex due to spin-orbital coupling phenomenon. The pair of peaks at ≈ 163.9 eV (2p_3/2_) and 164.9 eV (2p_1/2_) can be attributed to the C–S–C covalent bonds, those at 165.4 and 166.4 eV to C-SH, and that at 168.6 eV is assigned to some oxidized sulphur (-C-SO_x_-C-, x = 2, 3) [23,24]. The XPS surface atomic percentages were also determined, at 97.1% (C 1s), 1.1% (O 1s), 1.1% (N 1s) and 0.5% (S 2p). 

**Figure 1:**
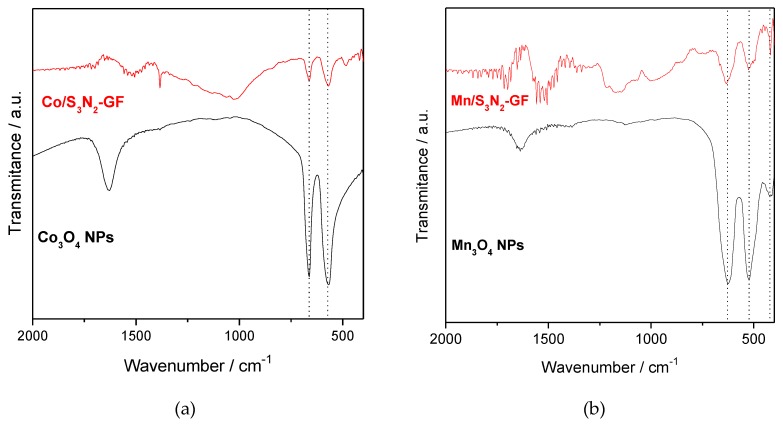
FTIR spectra of Co/S_3_N_2_–GF (**a**) and Mn/S_3_N_2_–GF (**b**) composites.

The initial assessment of the composite ECs was performed by FTIR and the spectra of Co/S_3_N_2_–GF and Mn/S_3_N_2_–GF are exhibited in Figure 1 (see Figure S2 for full spectra). For comparison, those of metal oxide NPs are also included. The presence of two bands at 575 and 662 cm^−1^ in the Co/S_3_N_2_–GF spectrum (Figure 1a) confirms the successful preparation of the desired composite. These two bands confirm the Co_3_O_4_ with spinel structure [25,26]. Similarly, the presence of two bands at 629 and 522 cm^−1^ in the Mn/S_3_N_2_–GF spectrum (Figure 1b) indicates that the Mn_3_O_4_ nanoparticles were prepared with spinel structure [21,27]. The composites also present the bands related to the S, N-doped GF (≈3591, 1534, 1325 and 1178 cm^−1^). Those at approximately 3591, 1534 and 1178 cm^−1^ correspond to the stretching vibrations of the OH groups, C=C and C–O/ C–S, respectively, while that at 1325 cm^−1^ corresponds to the C–N stretching vibration [28,29].

**Figure 2:**
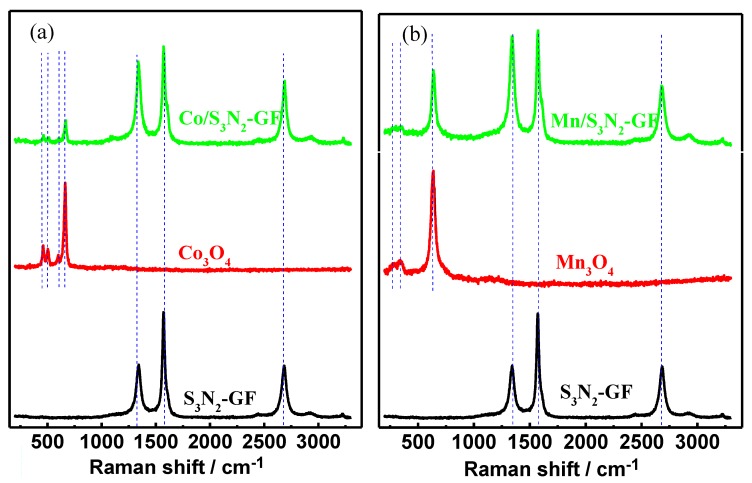
Raman spectra of Co/S_3_N_2_–GF (**a**) and Mn/S_3_N_2_–GF (**b**) composites.

Raman spectra of the prepared composites and of S_3_N_2_–GF, Co_3_O_4_ and Mn_3_O_4_ nanoparticles are depicted in Figure 2. The spectrum of the S_3_N_2_–GF shows peaks at 1336 cm^−1^ (D band), 1570 cm^−1^ (G band), 2684 cm^−1^ (2D band) and a smaller one at 2926 cm^−1^ (D + G band). Graphene doping shifts the Fermi level away from the Dirac point, decreasing the probability of charge carrier recombination [30]. The reduced recombination increases and sharpens the G band as can be seen in Figure 2. An increased electron (e^−^) concentration results in a decrease in the 2D band peak position, with an expanded crystal lattice, decreased Raman phonons and asymmetry in the doping effect of the G band peak position [31,32]. The presence of Co_3_O_4_ nanoparticles in the Co/S_3_N_2_–GF composite (Figure 2a) is confirmed by the peaks assigned to the Raman active modes of the Co_3_O_4_ structure at 671 (A_1g_), 603 (F_2g_), 507 (F_2g_) and 466 cm^−1^ (E_g_) [33,34]. The presence of the Raman bands corresponding to the doped carbon material is also observed (1342, 1573 and 2694 cm^−1^). The slight shift in the peak positions with respect to the undoped material suggests the interaction between the doped GF and the nanoparticles. 

The successful in situ preparation of Mn_3_O_4_ nanoparticles with spinel structure in the presence of S_3_N_2_–GF (Figure 2b) is also confirmed through the existence of Raman peaks at 640 (A_1g_), 351 (T_2g_) and 273 cm^−1^ (E_g_). The peaks assigned to the D, G and 2D modes are also observed at 1342 cm^−1^, 1575 cm^−1^ and 2682 cm^−1^, respectively.

**Figure 3:**
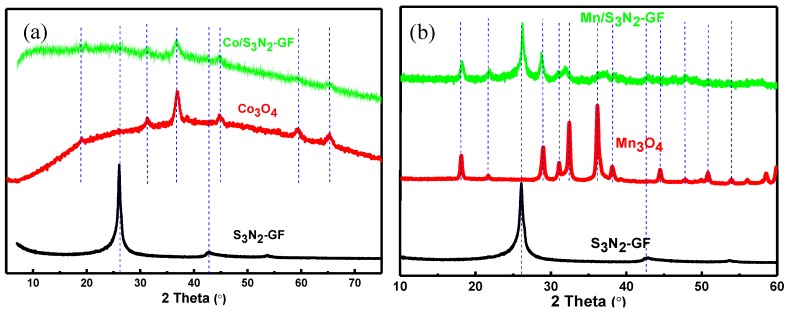
X-ray diffraction spectra of Co/S_3_N_2_–GF (**a**) and Mn/S_3_N_2_–GF (**b**) and the individual elements (S_3_N_2_–GF and metal oxide NPs).

XRD analysis was also carried out and the patterns can be observed in Figure 3. The XRD pattern of S_3_N_2_–GF shows diffraction peaks at 25°, 43° and 54°, corresponding to the 002, 100 and 101 XRD crystal planes, respectively, which can be indexed to the hexagonal crystalline graphite (JCPDS No. 41-1487) [35,36]. The XRD pattern of Co_3_O_4_ NPs (Figure 3a) shows the typical diffraction peaks indexed to the (111), (220), (311), (222), (400), (511) and (440) crystal planes of face-centred-cubic phase of the Co_3_O_4_ spinel structure at 2θ = 19°, 31°, 37°, 38°, 45°, 59° and 65° (JCPDS No. 42-1467) [37]. These results are in accordance with previously reported results [21,37,38]. The Co/S_3_N_2_–GF XRD patterns present one peak at 2θ = 26º assigned to the (002) XRD plane of stacked graphene layers, and those assigned to Co_3_O_4_ NPs. In Figure 3b are presented the XRD pattern of Mn_3_O_4_ NPs and corresponding Mn/S_3_N_2_–GF composite. In both patterns peaks can be observed at 2θ ≈ 18°, 29°, 31°, 32°, 36°, 38°, 44°, 51°, 54°, 56°, 58° and 60º, indexed to (101), (112), (200), (103), (211), (004), (220), (105), (312), (303) and (321) XRD crystal planes of a body-centred-cubic phase/structure, respectively (ICDD PDF card no.04-004-864). The presence of these peaks confirms the existence of Mn_3_O_4_ NPs with spinel structure [39]. The XRD results validate the results obtained by Raman analysis confirming in situ preparation of metal oxide NPs in the presence of S_3_N_2_–GF. Additionally, the particle size of Co_3_O_4_ and Mn_3_O_4_ nanoparticles (pristine and in the presence of S_3_N_2_–GF) was estimated using Scherrer equation [40]. The Scherrer equation was used on the most prominent peak (2θ = 27º for Co_3_O_4_ and Co/S_3_N_2_–GF, 2θ = 36º for Mn_3_O_4_ and 2θ = 29º for Mn/S_3_N_2_–GF) leading to estimated particle size of 23.0, 13.1, 26.2 and 28.6 nm for Co_3_O_4_, Co/S_3_N_2_–GF, Mn_3_O_4_ and Mn/S_3_N_2_–GF, respectively. 

**Figure 4:**
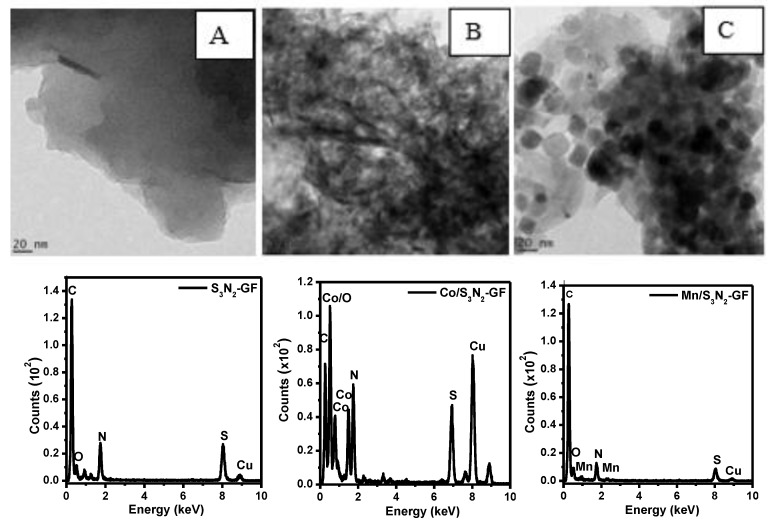
Transmission electron micrographs of S_3_N_2_–GF (**A**), Co/S_3_N_2_–GF (**B**) and Mn/S_3_N_2_–GF (**C**) with their corresponding EDS spectra.

The morphology of S_3_N_2_–GF and of both composites (Mn/S_3_N_2_–GF and Co/S_3_N_2_–GF) was evaluated using TEM (Figure 4). The TEM image of S_3_N_2_–GF (Figure 4a) shows graphene sheets which appear transparent and clear, consistent with literature reports [41,42]. The TEM image of Co/S_3_N_2_–GF suggests that spherical Co_3_O_4_ nanoparticles, which have been formed, agglomerate onto the surface of S_3_N_2_–GF sheets (Figure 4b). For the Mn/S_3_N_2_–GF composite, the TEM images show a section with dispersed tetragonal-shaped Mn_3_O_4_ NPs on the surface of S_3_N_2_–GF and another with some agglomeration (Figure 4c). It can also be seen on the TEM micrographs that the Mn_3_O_4_ nanoparticles formed comparably larger particles on the surface of S_3_N_2_–GF sheets when compared with the Co_3_O_4_ nanoparticles. The energy dispersive spectroscopy (EDS) analysis also suggests the presence of Mn_3_O_4_ and Co_3_O_4_, through the observation of Mn and Co species on the spectra. The presence of Cu on the spectra arises from the copper grids used as support during TEM analysis.

### 3.2. Electrochemical Performance of the Electrocatalysts towards ORR

**Figure 5:**
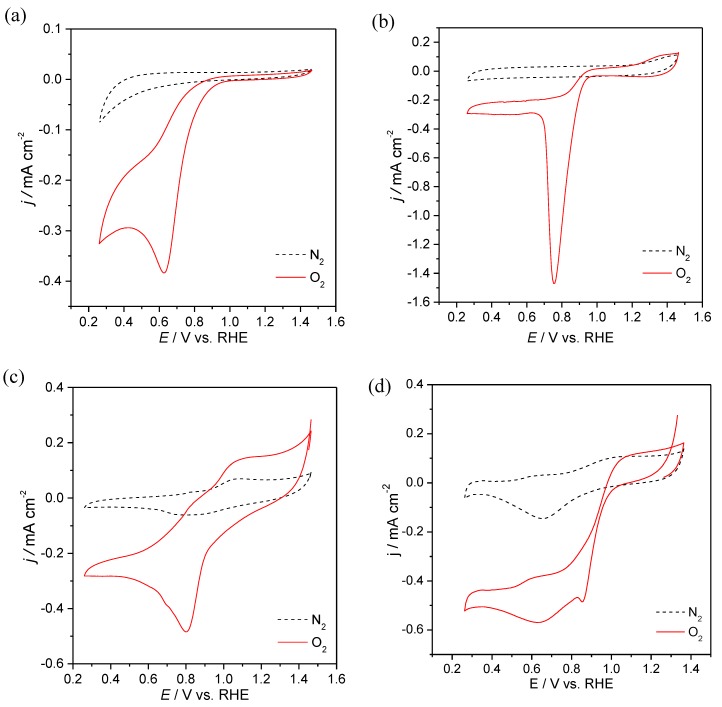
CVs in N_2_-saturated (dash line) and O_2_-saturated (full line) 0.1 mol dm^−3^ KOH solution at 0.005 V s^−1^ for S_3_N_2_–GF (**a**), Co/S_3_N_2_–GF (**b**), Mn/S_3_N_2_–GF (**c**) and commercial Pt/C (20wt%) modified electrodes (**d**).

The three materials prepared (S_3_N_2_–GF, Co/S_3_N_2_–GF and Mn/S_3_N_2_–GF) were evaluated as ORR electrocatalysts using cyclic voltammetry (CV) in nitrogen- and oxygen-saturated solutions (0.1 mol dm^−3^ KOH). The CVs can be observed in Figure 5. In the absence of oxygen, no electrochemical processes are observed for S_3_N_2_–GF and Co/S_3_N_2_–GF, whereas for Mn/S_3_N_2_–GF a redox pair with very low intensity can be observed (*E*_pc_ = 0.85 V and *E*_pa_ = 1.07 V vs. RHE). This pair of peaks can be attributed to manganese redox processes [21,43]. On the other hand, when oxygen is present, all composites show an irreversible ORR peak at *E*_pc_ = 0.63, 0.76 and 0.80 V vs. RHE for S_3_N_2_–GF, Co/S_3_N_2_–GF and Mn/S_3_N_2_–GF, respectively. In the same experimental conditions, for Pt/C (20 wt. %) the ORR peak was observed at *E*_pc_ = 0.86 V vs. RHE. 

**Figure 6:**
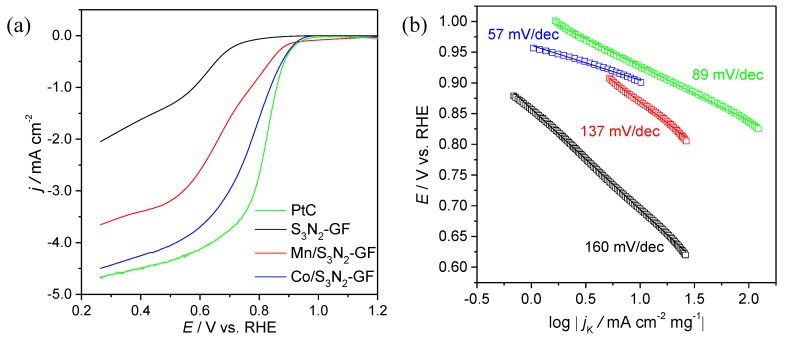
ORR LSV curves obtained in 0.1 mol dm^−3^ KOH saturated with O_2_ for Pt/C, S_3_N_2_–GF, Co/S_3_N_2_–GF and Mn/S_3_N_2_–GF at 1600 rpm and 0.005 V s^−1^ (**a**) and the corresponding ORR Tafel plots (**b**).

All composites were further studied by linear sweep voltammetry (LSV) in 0.1 mol dm^−3^ KOH in both N_2-_ and O_2_-saturated solutions. Figure 6a shows the LSV for Pt/C, S_3_N_2_–GF, Co/S_3_N_2_–GF and Mn/S_3_N_2_–GF in O_2_-saturated solution (after the subtraction of LSV in N_2_-saturated solution) and the main parameters determined are in Table 1. It can be clearly seen that the introduction of cobalt and manganese oxide nanoparticles into the S, N-doped GF leads to an improvement of the ORR features, not only in terms of diffusion-limiting current density (*j*_L_) values, but also onset potentials. The first duplicates from S_3_N_2_–GF (−2.05 mA cm^−2^) to Co/S_3_N_2_–GF and Mn/S_3_N_2_–GF (−4.50 and −3.66 mA cm^−2^, respectively), while the *E*_onset_ values shift 140 and 120 mV, respectively, towards more positive potentials. The onset potentials of both composites are similar to the value obtained for Pt/C and Co/S_3_N_2_–GF present comparable *j*_L_ value to that obtained for Pt/C. However, Mn/S_3_N_2_–GF shows a lower *j*_L_ value than Pt/C (less 20%). As evidenced by LSV results, Co/S_3_N_2_–GF displays the highest ORR activity among the three composites prepared, which is reflected by its having the most positive *E*_onset_ and highest *j*_L_ value.

**Figure 7:**
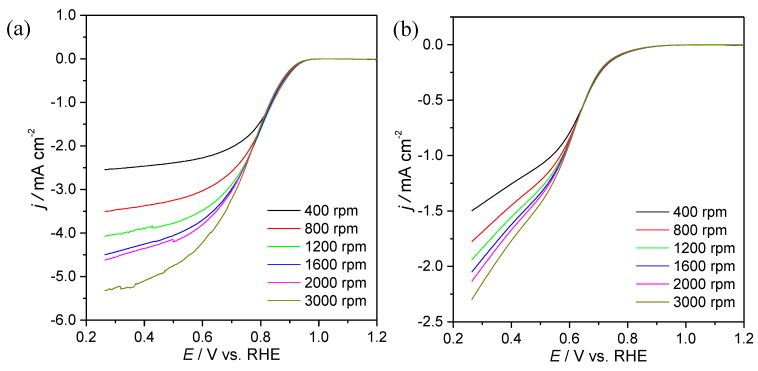

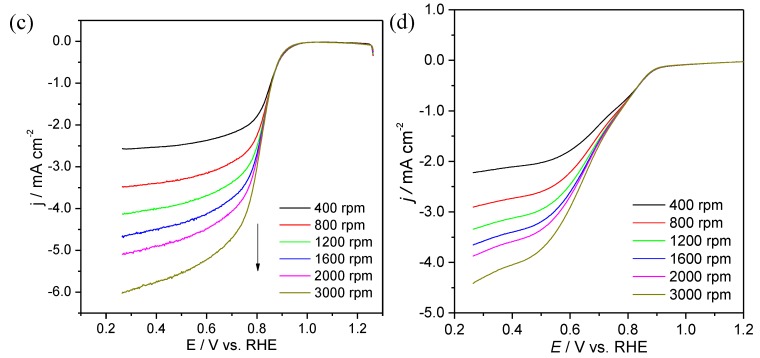
ORR polarization curves at different rotation rates in O_2_-saturated 0.1 mol dm^−3^ KOH solution at 0.005 V s^−1^ for S_3_N_2_–GF (**a**), Co/S_3_N_2_–GF (**b**), Mn/S_3_N_2_–GF (**c**) and commercial Pt/C (20wt%) modified electrodes (**d**).

**Figure 8:**
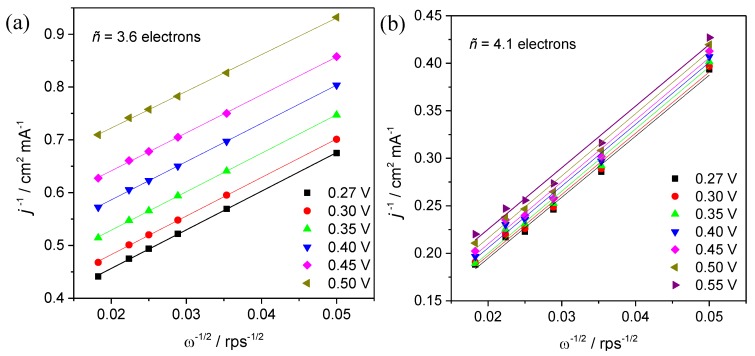

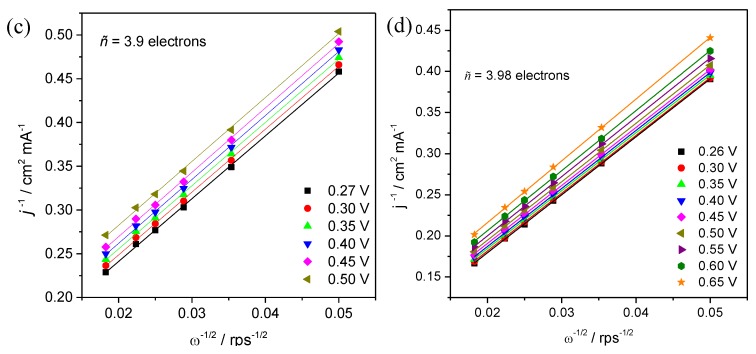
Koutecky–Levich (KL) plots obtained from the data in Figure 7 for S_3_N_2_–GF (**a**), Co/S_3_N_2_–GF (**b**), Mn/S_3_N_2_–GF (**c**) and commercial Pt/C (20wt%)-modified electrodes (**d**).

LSVs at different rotation speeds and quantitative Koutecky–Levich (KL) plots were obtained to gather more information about the ORR kinetics of these composites. Figure 7 shows the LSV in O_2-_saturated solution (after the subtraction of LSV in N_2_-saturated solution) while the corresponding KL plots can be observed in Figure 8. Both the composites prepared and Pt/C electrocatalyst show a first order ORR in relation to the concentration of oxygen dissolved with the KL plots presenting good linearity with lines showing similar slopes in the potential range scanned for each material. This suggests that the applied potential does not have a significant influence on the number of electrons transferred per oxygen molecule (*n*_O__2_). In fact, the *n*_O__2_ values estimated were almost constant with *n*_O__2_ = 4.0, 3.6, 4.1 and 3.9 for Pt/C, S_3_N_2_–GF, Co/S_3_N_2_–GF and Mn/S_3_N_2_–GF, respectively. These values show that the ORR process at these electrocatalysts seems to proceed via a direct pathway (direct reduction of oxygen to water) involving four electrons. In the fuel cell industry, this is the preferred pathway, since it directly produces OH^−^ ions as the final product without the formation of OOH^−^ ions. 

The results obtained for the Co/S_3_N_2_–GF and Mn/S_3_N_2_–GF composites are similar or better than others reported for similar compounds. For example, Duan et al. reported the application of Mn_3_O_4_ nanoparticles on nitrogen-doped graphene (Mn_3_O_4_@N–GF) [43] and on N-doped reduced graphene oxide (Mn_3_O_4_/N-rGO) [44] as ORR electrocatalysts with 0.87 ≥ *E*_onset_ ≥ 0.83 V and *n*_O__2_ = 3.8. Wang and co-workers have also successfully applied a Co_3_O_4_@g-C_3_N_4_/NG electrocatalyst for ORR, reaching an *n*_O__2_ of 3.9 and a *j*_L_ of −5.0 mA cm^−2^; however, the preparation of the electrocatalysts was time consuming and involved harsh conditions (higher temperatures) [10]. 

The excellent ORR performance of the as-prepared composites can be attributed to nitrogen and sulphur dual-doping, introduced asymmetry charge and spin density, and strong coupling between Co_3_O_4_ or Mn_3_O_4_ nanoparticles and doped graphene flakes. The unbalanced charge distribution resulting from nitrogen and sulphur dual-doping is considerably favourable to oxygen adsorption, while a strong bonding between doped graphene flakes and Co_3_O_4_ or Mn_3_O_4_ nanoparticles (NPs) can facilitate the electron transfer, and also assures a good durability. 

Tafel plots were obtained from the LSV curves at 1600 rpm and are depicted in Figure 6b. The Pt/C electrocatalysts presents an ORR Tafel slope of 89 mV dec^−1^ while for S_3_N_2_–GF, Co/S_3_N_2_–GF and Mn/S_3_N_2_–GF composites the values obtained were 160, 57 and 137 mV dec^−1^. These results suggest that for Co/S_3_N_2_–GF the global reaction rate is ruled by the conversion of intermediate surface adsorbed specie MOO- to MOOH with M representing an empty site on the surface of EC. For S_3_N_2_–GF and Mn/S_3_N_2_–GF the rate is defined by the consumption of MOOH species or by the first discharge step [45]. Additionally, the fact that the Co/S_3_N_2_–GF composite presents a lower Tafel slope than Pt/C suggests that it can easily adsorb oxygen molecules onto its surface and activate it, boosting the ORR electrocatalytic performance.

**Figure 9:**
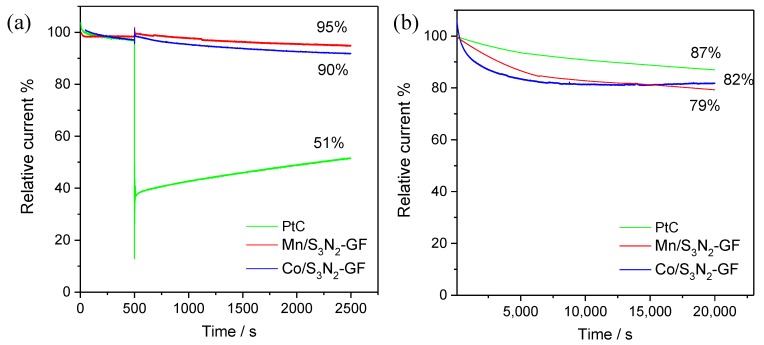
Chronoamperometric responses at *E* = 0.50 V vs. RHE and 1600 rpm of Pt/C, Co/S_3_N_2_–GF and Mn/S_3_N_2_–GF in 0.1 mol dm^−3^ O_2_-saturated KOH with the addition of 0.5 mol dm^−3^ methanol at *t* = 500 s (**a**) and without any addition for 20,000 s (**b**).

The application of electrocatalysts as cathodes in fuel cells powered by methanol is highly dependent on their tolerance to methanol crossover. The evaluation of potential electrocatalysts’ methanol tolerance is of extreme importance since in this type of fuel cells, the fuel (methanol) can permeate from the anode to the cathode through the polymer membrane. This will cause a critical decay in the EC performance as the active sites will be used for methanol oxidation contributing to a progressive CO poisoning leading to a continuous decrease in active sites for ORR. To evaluate the methanol crossover effect, a chronoamperometric test was performed in 0.1 mol dm^−3^ KOH saturated with O_2_ and the results are presented in Figure 9a.

The addition of methanol causes a current decrease of ≈ 49% when Pt/C is used; however, for Co/S_3_N_2_–GF and Mn/S_3_N_2_–GF, its influence is almost insignificant, leading to current retentions of 90% and 95%, respectively. These results suggest a greater selectivity of the prepared composites towards ORR compared to methanol oxidation which constitutes a huge advantage when developing electrocatalysts for direct methanol fuel cells. 

Another crucial issue when developing ORR electrocatalysts is their durability. In order to evaluate the Co/S_3_N_2_–GF and Mn/S_3_N_2_–GF stability, a chronoamperometric test was performed for 20,000 s in O_2_-saturated KOH at 0.50 V and 1600 rpm. Figure 9b shows the obtained results (Pt/C was included for comparison) and it can be seen even though Pt/C shows higher stability (87% current retention), the prepared electrocatalysts present very similar values of current retention (79% and 82%) suggesting good stability. 

## 4. Conclusions 

The synthesis of metal oxide (Mn_3_O_4_ and Co_3_O_4_) impregnated the S_3_N_2_–GF electrocatalyst with enhanced electrochemical properties towards ORR is here reported using a simple, scalable and cost-effective method. The structural characterization of the materials showed the preservation of the Mn_3_O_4_ and Co_3_O_4_ spinel structures. All prepared nanocomposites displayed superior overall ORR electrocatalytic activity in the alkaline medium with Co/S_3_N_2_–GF and Mn/S_3_N_2_–GF presenting the most promising results, with *E*_onset_ values of 0.91 and 0.89 V vs. RHE and *j*_L_ values of −4.50 and −3.66 mA cm^−2^, respectively. The improved performance of metal oxide-containing composites was attributed to the coupling between Co_3_O_4_ or Mn_3_O_4_ nanoparticles and nitrogen and sulphur dual-doped graphene flakes. Furthermore, these two composites revealed an excellent tolerance to methanol and good stability. This work contributed to an efficient, simple, scalable and low-cost procedure for the development of efficient and naturally abundant ORR electrocatalysts.

## Figures and Tables

**Table 1:** Onset potentials (*E*_onset_), diffusion-limiting current density values (*j*_L,0.26 V,1600 rpm_) and Tafel slopes determined from the ORR LSV curves in 0.1 mol dm^−3^ KOH and the number of electrons transferred for each O_2_ molecule.

Sample	*E*_onset_ (5%Total)	*E*_onset_ (*j* = 0.1mAcm^−2^)	*j*_L_ (mA cm^−2^)	Tafel (mV dec^−1^)	*ñ*O_2_
Pt/C	0.91	0.94	−4.67	89	4.0
S_3_N_2_–GF	0.77	0.77	−2.05	164	3.6
Co/S_3_N_2_–GF	0.91	0.93	−4.50	57	4.1
Mn/S_3_N_2_–GF	0.89	0.89	−3.66	137	3.9

## Supplementary Materials

The following are available online at https://www.mdpi.com/1996-1944/13/7/1562/s1, Figure S1 shows the deconvoluted high resolution XPS spectra of S3N2–GF: C 1s (a), O 1s (b), N 1s (c) and S 2p (d) and Figure S2 shows the full FTIR spectra.
