# Supplementary material for: Metal Oxide (Co3O4 and Mn3O4) Impregnation into S, N-doped Graphene for Oxygen Reduction Reaction (ORR)
Source: Materials (Basel). 2020 Mar 28;13(7):1562. doi: 10.3390/ma13071562 (PMC7177977; doi:10.3390/ma13071562)
Supplement: Supplementary file 1 [file materials-13-01562-s001.pdf]

materials

Supplementary Materials: Metal Oxide Impregnation (Co_3O_4 and Mn_3O_4) into S, N-doped Graphene for Oxygen Reduction Reaction (ORR)

Penny Mathumba, Diana M. Fernandes *, Renata Matos, Emmanuel I. Iwuoha and Cristina Freire

1. Experimental

1.1. Materials and Solvents

For the electrocatalysts preparation: graphene flakes (Graphene Technologies, Novato, USA, Lot GTX- 7/6–10.4.13), 2-5-dimercapto-1,3,4-thiadiazole (S_3N_2, Sigma-Aldrich), manganese (II) chloride tetrahydrate ($MnCl_2.4H_2O$, Sigma-Aldrich, Algés, Portugal), cobalt (II) chloride hexahydrate ($CoCl_2$, Sigma-Aldrich), 1-amino-2-propanol (MIPA, 93%, Aldrich) and absolute ethanol (Fisher Scientific, USA)

For the electrochemical studies: potassium hydroxide (99.99%, Sigma-Aldrich), platinum nominally 20% on carbon black (Pt/C 20 wt.%, HiSPEC® 3000, Alfa Aesar, Kandel, Germany), Nafion (5 wt.% in lower aliphatic alcohols and water, Aldrich), isopropanol (99.5%, Aldrich), methanol (anhydrous, VWR) and hydrogen peroxide solution (30 wt.% in water, ACS reagent, Sigma-Aldrich). Ultrapure water (18.2 MΩ cm at 25 ºC, Millipore, Algés, Portugal) was used throughout the experiments

1.2. Materials Preparation

The Co_3O_4 nanoparticles were prepared through a co-precipitation procedure where a 3.0 mol dm^{-3} solution of MIPA was added to 50 mL of an aqueous solution of $CoCl_2 \cdot 6H_2O$ (5.0 mmol), at a rate of 50 mL h^{-1}, until *pH* = 10 was reached. The reaction mixture was stirred for 24 h, at room temperature. The resulting material was filtered, thoroughly washed with water and ethanol, and dried under vacuum. Then, the powder was calcined under air, at 250 ºC for 3 h. The Mn_3O_4 nanoparticles were prepared through a similar procedure: MIPA was added to 100 mL of an aqueous solution of $MnCl_2 \cdot 4H_2O$ (11.0 mmol), until *pH* = 10 and the mixture was stirred for 24 h at 80 ºC under reflux. The calcination was performed under air at 300 ºC for 5 h.

1.3. Physicochemical Characterization

X-ray photoelectron spectroscopy (XPS) measurements were performed at the Centro de Materiais da Universidade do Porto (CEMUP), Portugal, on A VG Scientific ESCALAB 200A spectrometer with non-monochromatized Al K*α* radiation (1486.6 eV) was used for X-ray photoelectron spectroscopy (XPS) measurements at CEMUP. Potential deviations induced by electric charge of the samples were corrected using the C 1s band at 284.6 eV as an internal standard. The analysis of XPS results was performed using the CasaXPS software for spectra deconvolution. Surface atomic percentages were calculated from the corresponding peak areas upon spectra deconvolution and using the sensitivity factors provided by the manufacturer.

The infrared spectra (Fourier transform infrared, FTIR) were acquired using a Jasco FT/IR-460 Plus spectrophotometer (64 scans, resolution of 4 cm^{-1}, in the range of 400 - 4000 cm^{-1}). The samples (0.2% of material) were dispersed in KBr pellets (spectroscopic grade, Merck).

The micro-Raman analysis was conducted in the backscattering configuration on a Jobin Yvon HR800 instrument (Horiba, Japan), using a 600 lines/mm grating and the 532 nm laser line from a Nd:YAG DPSS laser (Ventus, Laser Quantum, U.K.). For the Rayleigh rejection, a pair of edge filters placed in series was used allowing Raman acquisition from 50 cm^{-1}. A 100× objective (spot size < 2 μm, numeric aperture = 0.9, Olympus, Japan) was used to focus the laser light onto the sample and

to collect the backscattered Raman radiation to be detected by a Peltier cooled (223 K) CCD sensor. The spectrometer was operated in the confocal mode, setting the iris to 300 μm.

Powder X-ray diffraction (XRD) analyses were performed at Instituto de Física dos Materiais da Universidade do Porto, IFIMUP (Porto, Portugal). XRD patterns were obtained with a Rigaku Smartlab X-ray Diffractometer, involving X-ray source CuKα (λ = 1.5418 Å; acceleration potential = 45 kV; current = 200 mA).

The surface morphology, particle size and size distribution of nanomaterial were examined using TEM microscopy, a Tecnai G2 F20X-Twin MAT 200 kV Field Emission Transmission Electron Microscope (FEI Eindhoven, Netherlands). Prior analysis the samples were dispersed in ethanol and thereafter deposited on copper grid for analysis under TEM microscopy.

1.4. ORR Electrochemical Tests

A potentiostat/galvanostat Autolab PGSTAT 302N (ecochimie B.V.), controlled by the NOVA v2.1 software was used for the CV and LSV tests. The experiments were conducted at room temperature using a conventional three-electrode compartment cell: 1) reference electrode - Ag/AgCl (3 mol.dm^{-3} KCl, Metrohm); 2) working electrode - glassy carbon rotating disk electrode (RDE) (3 mm of diameter, Metrohm); 3) auxiliary electrode - carbon rod (2 mm of diameter, Metrohm).

Before modification, a cleaning procedure was performed to the RDE with diamond polishing pastes of 6, 3 and 1 μM (Buehler) on a microcloth pad (BAS), followed by washing with ultra-pure water (18.2 MΩ cm at 25°C, Millipore). For the RRDE, the cleaning procedure was performed only with 0.3 μm alumina powder (MicroPolish Alumina, Buehler) in order to prevent damage of Pt ring. The RDE was then modified through the deposition of a 5 μL drop of the selected EC dispersion onto its surface and allowing it to dry under a flux of air. The ECs dispersion was prepared as follows: 1 mg of selected material or Pt/C were mixed with isopropanol/water/Nafion solvent mixture (125/125/20 μL) and dispersed using an ultrasonic bath for at least 15 min. Electrochemical tests were carried out in N_2- or O_2-saturated KOH (0.1 mol dm^{-3}). To achieve this, the electrolyte was degassed for 30 min with the selected gas.

For the evaluation of ORR performance, both the CV and LSV measurements were performed between E_P = 0.26 and 1.46 V vs. RHE at 0.005 V s^{-1}. Additionally, rotation speeds in the range 400 - 3000 rpm were used for the LSV experiments. For the chronoamperometry (CA) tests a rotation speed of 1600 rpm for 20,000 s at a potential = 0.55 V vs. RHE was used. Tolerance to methanol was assessed by CA at E = 0.55 V vs. RHE and 1600 rpm for 2500 s.

The effective ORR current was obtained by subtracting the current obtained in N_2-saturated electrolyte from that obtained in O_2-saturated electrolyte.

Even though the potential were measured against the Ag/AgCl reference electrode, these were converted to the reversible hydrogen electrode (RHE) using the Eq. 1 for a proper comparison with the literature results.

$$E_{(RHE)} = E_{(Ag/AgCl)} + 0.059\ pH + E^{o}_{(Ag/AgCl} \quad (1)$$

where $E_{(RHE)}$ is the potential vs. RHE, $E^{o}_{(Ag/AgCl)}$ = 0.1976 V (25 ºC) and $E_{(Ag/AgCl)}$ is the potential measure vs. Ag/AgCl.

Onset potential (E_{onset}) is defined as the potential at which the reduction of O_2 begins. According to literature, the E_{onset} can be determined by different methods and is generally assume as the potential at which the ORR current is 5% of the diffusion-limiting current density, or it can be calculated as the potential at which the slope of the voltammogram exceeds a threshold value (= 0.1 mA cm^{-2}V^{-1}) [1]. Here we considered the first method.

The kinetic parameters and the number of electrons transferred per O_2 molecule (n_{O2}) in the oxygen reduction reaction were determined using the following Koutecky–Levich (KL) equations [1,2]:

$$\frac{1}{j} = \frac{1}{j_L} + \frac{1}{j_k} = \frac{1}{B\omega^{1/2}} + \frac{1}{j_k} \quad (2)$$

$$B = 0.2\, n_{O2}\, F\, (D_{O2})^{2/3}\, v^{-1/6}\, C_{O2} \quad (3)$$

Here, j is the current density measured, j_L and j_k are the diffusion-limiting and kinetic current densities, ω is the angular velocity, F is the Faraday constant (96 485 C mol^{-1}), D_{O2} is the O_2 diffusion coefficient (1.95×10^{-5} cm^2 s^{-1}), v is the electrolyte kinematic viscosity (0.008977 cm^2 s^{-1}), C_{O2} is the O_2 bulk concentration (1.15×10^{-3} mol dm^{-3}). For rotation speeds in rpm is adopted a constant of 0.2.

Tafel plots were obtained after the measured LSV currents were corrected for diffusion to yield the corresponding kinetic current values. The j_L parameter, obtained through the combination of Eq. 2 and 3, was used to make the mass transport correction. The values of j_k obtained were normalized for the total deposited mass of EC.

Rotating ring disk electrode (RRDE) measurements were also performed in the O_2-saturated KOH solution in order to obtain a more in-depth insight into the ORR electrocatalytic activity of the ECs. The H_2O_2 yields were determined from the ring and disk currents (i_R and i_D, respectively), and the current collection efficiency of the Pt ring (N = 0.25, in this case) using Equation (1) [1]:

$$\%\ H_2O_2 = 200 \times \frac{i_R/N}{i_D + i_R/N} \quad (4)$$

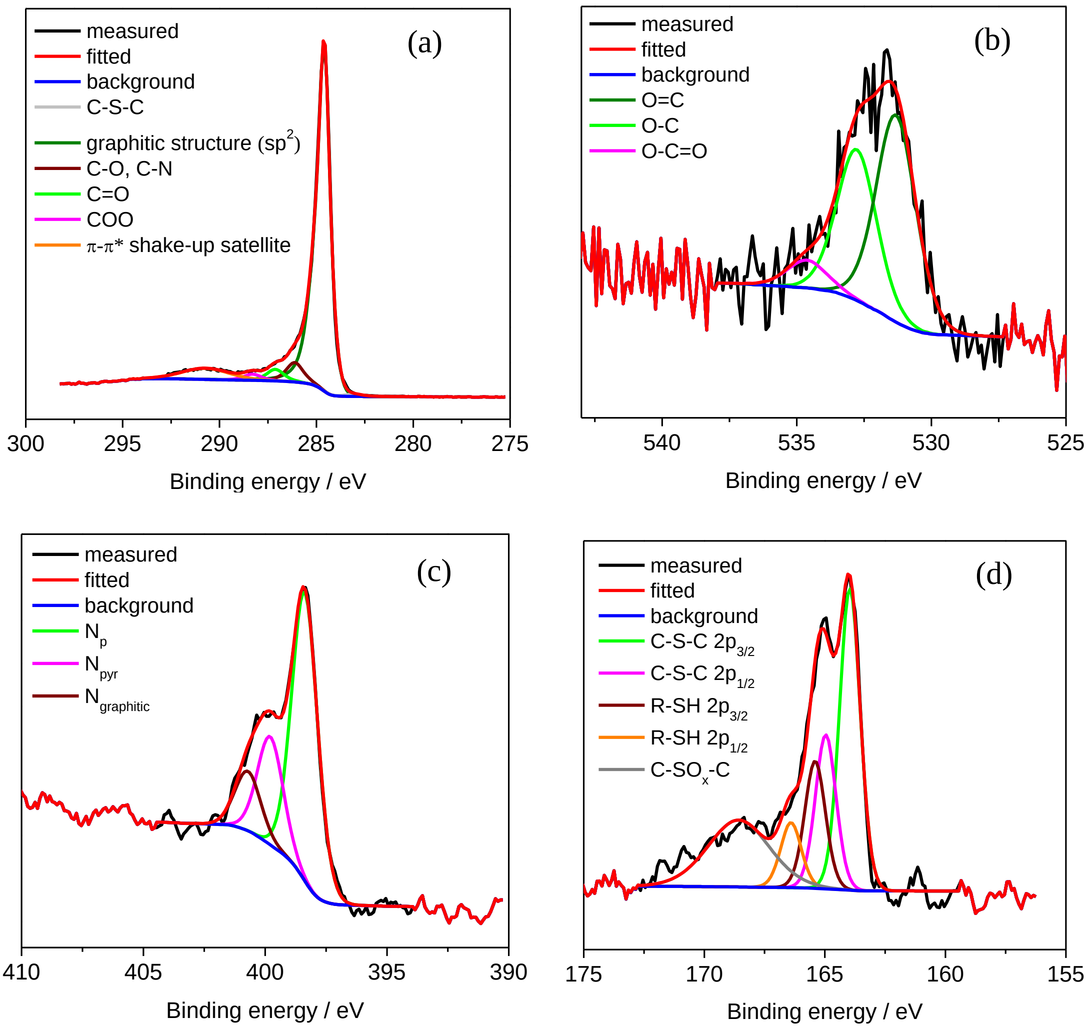

Figure S1. Deconvoluted high resolution XPS spectra of S_3N_2-GF: C 1s (a), O 1s (b), N 1s (c) and S 2p (d).

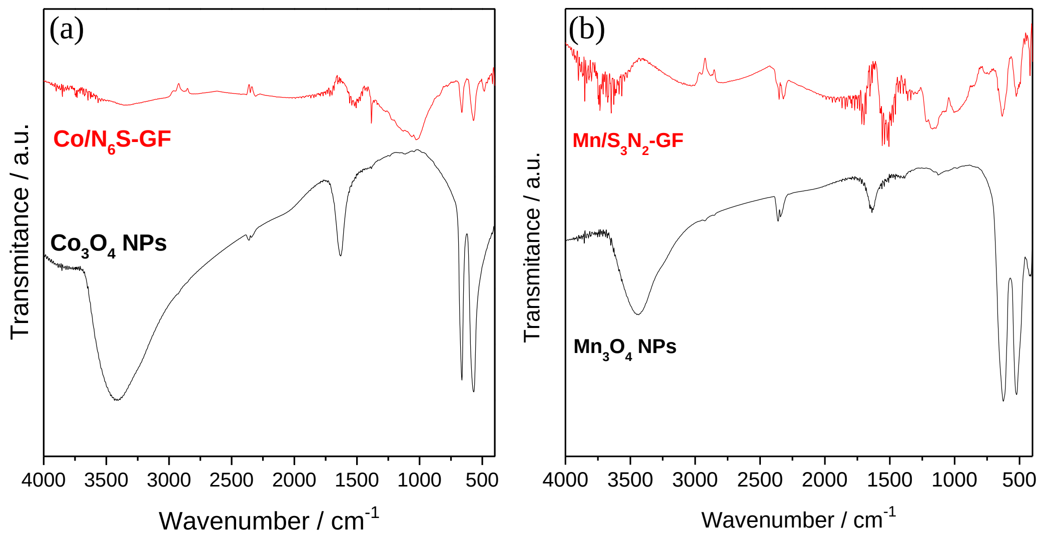

Figure S2. FTIR spectra of Co/S_3N_2-GF (a) and Mn/S_3N_2-GF (b) composites.

References

1. Fernandes, D. M.; Mathumba, P.; Fernandes, A. J. S.; Iwuoha, E. I.; Freire, C., Towards efficient oxygen reduction reaction electrocatalysts through graphene doping. *Electrochim. Acta.* **2019,** *319*, 72–81.
2. Stacy, J.; Regmi, Y. N.; Leonard, B.; Fan, M. H., The recent progress and future of oxygen reduction reaction catalysis: A review. *Renew. Sust. Energ. Rev.* **2017,** *69*, 401–414.

© 2020 by the authors. Licensee MDPI, Basel, Switzerland. This article is an open access article distributed under the terms and conditions of the Creative Commons Attribution (CC BY) license (http://creativecommons.org/licenses/by/4.0/).
